# LncRNA TUG1 alleviates sepsis-induced acute lung injury by targeting miR-34b-5p/GAB1

**DOI:** 10.1186/s12890-020-1084-3

**Published:** 2020-02-22

**Authors:** Nan Qiu, Xinmei Xu, Yingying He

**Affiliations:** Department of Emergency Internal Medicine, Guizhou Provincial People’s Hospital, Guiyang City, No. 1 Baoshan South Road, Guiyang City, Guizhou Province China

**Keywords:** Acute lung injury, Apoptosis, miRNA, GAB1, Sepsis, TUG1, Inflammation

## Abstract

**Background:**

Sepsis-induced acute lung injury (ALI) is a clinical syndrome characterized by the injury of alveolar epithelium and pulmonary endothelial cells. This study aimed to investigate the regulation of long noncoding RNA (lncRNA) taurine up-regulated gene 1 (TUG1) in a murine ALI model and in primary murine pulmonary microvascular endothelial cells (PMVECs) stimulated with lipopolysaccharide (LPS).

**Methods:**

Adult C57BL/6 mice were intravenously injected with or without TUG1-expressiong adenoviral vector or control vector 1 week before the establishment of ALI model. PMVECs were transfected with TUG1-expressiong or control vectors followed by LPS stimulation. MiR-34b-5p was confirmed as a target of TUG1 using dual-luciferase reporter assay. GRB2 associated binding protein 1 (GAB1) was confirmed as a downstream target of miR-34b-5p using the same method. In the rescue experiment, PMVECs were co-transfected with TUG1-expressing vector and miR-34b-5p mimics (or control mimics) 24 h before LPS treatment.

**Results:**

ALI mice showed reduced levels of TUG1, pulmonary injury, and induced apoptosis and inflammation compared to the control group. The overexpression of TUG1 in ALI mice ameliorated sepsis-induced pulmonary injury, apoptosis and inflammation. TUG1 also showed protective effect in LPS-treated PMVECs. The expression of MiR-34b-5p was negatively correlated with the level of TUG1. TUG1-supressed apoptosis and inflammation in LPS-stimulated PMVECs were restored by miR-34b-5p overexpression. GAB1 was inversely regulated by miR-34b-5p but was positively correlated with TUG1 expression.

**Conclusion:**

TUG1 alleviated sepsis-induced inflammation and apoptosis via targeting miR-34b-5p and GAB1. These findings suggested that TUG1 might be served as a therapeutic potential for the treatment of sepsis-induced ALI.

## Background

Sepsis is a life-threatening condition caused by an excessive immune response to pathogen-induced infections [1]. It has become one of the leading predisposing clinical factors associated with the incidence of acute lung injury (ALI), a severe syndrome comprising a wide variety of acute respiratory failure disorders [2]. During the progression of sepsis-induced ALI, the upregulation of inflammatory and apoptotic pathways lead to the disruption of alveolar epithelial cells, the increase of epithelial permeability and the influx of edema fluid into the alveolar space [3]. It has been reported that persistently increased plasma levels of proinflammatory cytokines, such as TNF-α and interleukin (IL)-6, were highly predicative of mortality in patients with ALI [4, 5]. One study also showed that the increase of pro-apoptotic proteins, such as Bcl-2-associated X protein (Bax), favored the extensive apoptosis of alveolar epithelial cells and epithelial injury in ALI [6]. These findings suggested that the strategies to modulate inflammatory and apoptotic pathways might provide new opportunities to ameliorate sepsis-induced ALI in humans.

Long noncoding RNAs (lncRNAs) are a class of endogenous RNAs longer than 200 nucleotides and lack of protein-coding capabilities [7]. Emerging evidence has shown that they play key regulatory and functional roles in disease-associated gene transcription and chromatin modification [8]. Many lncRNAs have been identified to enhance or suppress inflammatory responses via regulating the expression of inflammatory mediators [9]. For instance, lncRNA E330013P06 contributed to a diabetes-induced proinflammatory phenotype via upregulating the level of proinflammatory genes and the formation of foam cells in macrophages [10]. LncRNA NKILA protected endothelial cells from inflammation by promoting the expression of kruppel like factor 4 and attenuating the transcriptional activity of nuclear factor kappa B [11]. Taurine up-regulated gene 1 (TUG1) is the lncRNA which was first detected in taurine-treated mouse retinal cells [12]. The downregulation of TUG1 has been demonstrated to inhibit cell proliferation in osteosarcoma and urothelial carcinoma cells [13, 14]. Knockdown of TUG1 in small cell lung cancer cells increased apoptosis and cell cycle arrest by regulating the expression of its target gene LIMK2b [15]. It was also shown that the overexpression of TUG1 alleviated cold-induced liver damage in mice via attenuating hepatocyte apoptosis and inflammation [16]. However, it remains unclear whether TUG1 has a regulatory role in sepsis-induced ALI.

In this study, we aimed to investigate the expression and regulation role of TUG1 in sepsis-induced ALI using a murine septic model and an in vitro cell culture model induced by lipopolysaccharide (LPS) stimulation. Further bioinformatic prediction analysis showed that miR-34b-5p was a potential downstream target of TUG1. The correlation between TUG1 level of miR-34b-5p were also examined. These findings suggested that TUG1 might be involved in the pathogenesis of sepsis-induced ALI via mediating the expression of its downstream target.

## Methods

### Mouse model of CLP-induced sepsis

Forty-eight adult male C57BL/6 mice (Charles River Laboratories, Beijing, China) were housed in a controlled environment with 12-h light-dark cycle, a temperature of 22–24 °C and a humidity of 60%. Mice were given ad libitum access to food and water. All experiments in this study were approved by the Animal Care and Use Committee of Guizhou Provincial People’s Hospital and performed in accordance with the Guide for the Care and Use of Laboratory Animals [17]. After one-week acclimatization, mice were randomly assigned into 4 groups: sham, CLP, CLP + Ad-GFP, and CLP + Ad-TUG1 (*n* = 12 per group). To establish the mouse model of sepsis-induced ALI, thirty-six mice underwent cecal ligation and puncture (CLP) surgery [18, 19]. Briefly, animals were anesthetized by intraperitoneal injection of 10% chloral hydrate (3 mL/kg, Sigma-Aldrich, St. Louis, USA) and fixed in supine position on the operating Table. A 0.4-cm longitudinal midline incision was made on the abdomen to expose the cecum. Then the exposed cecum was ligated at 1 cm from the tip using 3–0 silk sutures and perforated once by a 20-gauge needle at 0.5 cm distal from the ligation. After gently squeezing the cecum to extrude a small amount of feces, the bowel was repositioned in the abdominal cavity. Then the abdominal musculature, peritoneum and skin were closed. Mice received subcutaneous injection of normal saline immediately following the surgery for fluid resuscitation. Sham-operated group underwent the same surgical procedure except for the ligation or puncture of the cecum. After CLP procedure, mice were monitored for survival. The mouse lung tissues were harvested immediately after death or euthanasia by sodium pentobarbital (10 mg/kg intraperitoneal body weight) at the end of the study.

### Preparation and delivery of adenoviral vectors in vivo

Adenoviral vectors containing the enhanced green fluorescent protein gene were purchased from Life Technologies (Shanghai, China). The TUG1 cDNA (or a negative control) was transferred to the Ad-TUG1 (or Ad-GFP) vector using Gateway™ LR Clonase II Enzyme Mix (Invitrogen, Carlsbad, USA) as previously described [20]. Then 20 μL of adenovirus solution (10^7^ particles/μL) were intravenously injected via the tail vein into designated group 1 week before the CLP operation.

### Histological analyses

The lung tissues of all mice were harvested immediately after death or at the end of the study (3-day post-operation). The same portion of the lung samples were used for histopathological examination. Paraffin-embedded tissues were cut into 5 μm sections, stained with hematoxylin and eosin (H&E), and observed under a light microscope (magnification 400×). The degree of lung injury was evaluated using the lung injury scoring as previously described [21]. Five randomly selected fields were scored per slide. To detect cell apoptosis, tissue sections were stained with TUNEL reagent (Roche, Basel, Switzerland) according to the manufacturer’s instructions and observed using a fluorescence microscope (magnification 400×). Sectioned tissue samples were also stained for caspase-3 antibody (#ab13847, Abcam, Cambridge, UK) using immunohistochemistry method (magnification 400×). The percentage of positively stained cells was calculated in 6 randomly selected fields.

### Patients samples

A total of 35 patients who were diagnosed with acute respiratory distress syndrome (ARDS) in our hospital were recruited and their blood samples were collected. The blood samples were also harvested from 68 healthy subjects. Each patient provided a written informed consent. All experimental protocols were approved by the Ethics Committee of the Guizhou Provincial People’s Hospital and performed following the World Medical Association Declaration of Helsinki [22]. The serums from all the samples were prepared and the serum level of TUG1 was measured using an enzyme-linked immunosorbent assay (ELISA) kit (Novus Biologicals, Centennial, USA).

### Cell culture and transfection

Primary murine pulmonary microvascular endothelial cells (PMVECs) were isolated and cultured in DMEM (Sigma-Aldrich) as previously described [23]. The adenoviral vectors expressing TUG1 or a non-specific control sequence were synthesized as mentioned above. When reached 70–80% confluency, PMVECs were transfected with 1 μg adenoviral vector expressing TUG1 or control adenoviral vector for 48 h using ViraPower™ Adenoviral Expression System (Invitrogen) according to the manufacturer’s instructions. Twenty-four hours after transfection, PMVECs were stimulated with 100 ng/mL LPS (Sigma-Aldrich). Six hours following LPS treatment, PMVECs were fixed, stained with TUNEL and DAPI (Thermo Fisher Scientific, Waltham, USA) for the detection of apoptosis. DAPI stains cell nucleus, recognizing both apoptotic and non-apoptotic cells. The apoptotic cells were detected with dual TUNEL and DAPI staining. The number of TUNEL-positive cells were counted in six randomly selected fields per slide using a fluorescence microscope (magnification, × 200). The miR-34b-5p mimics and its corresponding control mimics (50 nM) were synthesized by GenePharm and transfected into PMVECs using Lipofectamine 2000 (Invitrogen). In rescue experiment, PMVECs were co-transfected with 1 μg adenoviral vector expressing TUG1 (or control adenoviral vector) and 50 nM miR-34b-5p mimics 24 h before LPS stimulation. PMVECs co-transfected with control adenoviral vector and control mimics were used as a control group.

### Dual-luciferase reporter assay

TUG1 3′-UTR fragment containing the putative binding site of miR-34b-5p was amplified and cloned into the downstream of luciferase gene in the pmirGlo vector (GenePharm, Shanghai, China). The mutant TUG1 3′-UTR was used to construct TUG1-MUT vector. HEK-293 cells were co-transfected with miR-34b-5p mimics (or miR mimics) and TUG1-WT (or TUG1-MUT) at 70–80% confluency. The synthesized GAB1 3′-UTR sequence (Ribobio, Guangzhou, China) or a mutant sequence were cloned into pmirGlo vectors (GenePharm) to construct luciferase reporters GAB1-WT and GAB1-MUT. HEK-293 T cells were co-transfected with GAB1-WT (or GAB1-MUT, 500 ng total DNA) and miR-34b-5p mimics (or miR mimics, 500 ng total DNA) at 70–80% confluency. The luciferase activities were assessed 48 h post transfection using Dual-Luciferase Reporter Assay System (Promega Biotech Co., Madison, USA).

### RNA immunoprecipitation (RIP) assay

The RIP assay was performed in PMVECs using mouse monoclonal anti-Argonaute2 (anti-Ago2) antibody (#SAB4200085, Sigma-Aldrich) as a positive control and an anti-IgG antibody (#R9255, Sigma-Aldrich) as a negative control using the Imprint® RNA Immunoprecipitation Kit (Sigma-Aldrich) as previously described [24]. Briefly, PMVECs were harvested, centrifuged and resuspended in RIP lysis buffer. Cell lysates were incubated with anti-Ago2 or anti-IgG overnight at 4 °C. The 40 μL Protein A magnetic beads were added to get the immunoprecipitation complex. Total RNA was extracted using GenElute™ Total RNA Purification Kit (Sigma-Aldrich). The relative RNA enrichment of TUG1 and miR-34b-5p were analyzed by quantitative real-time PCR.

### Quantitative real-time PCR (qRT-PCR)

Mouse lung tissue samples and PMVECs were harvested at the end of the study. Target miRNA was isolated from using mirVana™ miRNA Isolation Kit (Invitrogen). The reverse transcription of miR-34b-5p was performed using All-in-One™ miRNA RT-qPCR Detection Kit (GeneCopoeia Inc., Rockville, USA). Total RNAs were extracted using TRIzol LS Reagent (Invitrogen) and reverse transcribed to cDNA using ReverTra Ace qPCR RT Kit (Toyobo, Osaka, Japan) according to the manufacturer’s instructions. Target genes were amplified using 7300 Real-Time PCR System (Thermo Fisher Scientific). The expression of miR-34b-5p and mRNAs were normalized by U6 and β-actin, respectively. The sequences of the primers were: miR-34b-5p: CGAGGCAGTGTAATTAGCTGATTGT; U6 forward: CTCGCTTCGGCAGCACA, U6 reverse: AACGCTTCACGAATTTGCGT; TNF-α forward: GGGGCCACCACGCTCTTCTGTC, TNF-α reverse: TGGGCTACGGGCTTGTCACTCG; IL-1β forward: CCAGGATGAGGACCCAAGCA, IL-1β reverse: TCCCGACCATTGCTGTTTCC; IL-6 forward: TAGCCGCCCCACACAGACAG, IL-6 reverse: GGCTGGCATTTGTGGTTGGG; β-actin forward: ATCACTGCCACCCAGAAGAC, β-actin reverse: TTTCTAGACGGCAGGTCAGG.

### Enzyme-linked immunosorbent assay (ELISA)

Total proteins extracted from the lung tissue homogenates and PMVECs lysates were analyzed for the levels of TNF-α, IL-1β, IL-6, IL-4 and IL-10 using ELISA kits followed the manufacturer’s instructions (MyBioSource Inc., San Diego, CA, USA). The absorbance at 450 nm was detected using a Power Wave Microplate Reader (Bio-TEK, USA).

### Western blot

Total proteins isolated from homogenized mouse lung tissues and PMVECs lysates were prepared in RIPA buffer with protease inhibitors, and normalized by protein content using bicinchoninic acid assay (Pierce, Rockford, USA). Proteins at 40–80 μg were separated on a 10% SDS-PAGE gel under reducing conditions and then transferred to polyvinylidene fluoride (PVDF) membranes (MilliporeSigma, Burlington, USA). After blocking, PVDF membranes were incubated with designated primary antibodies (Abcam, Cambridge, UK) at 4 °C for 12 h: Bax (1:2000, #ab32503), B-cell lymphoma 2 (Bcl-2, 1:800, #ab59348), cleaved poly ADP ribose polymerase (cleaved PARP, 1:2000, #ab32064), cleaved caspase-3 (1:1000, #ab49822), GRB2 associated binding protein 1 (GAB1, 1:800, #ab59362), and β-catenin (1:2000, #ab32572). Then PVDF) membranes were incubated with goat anti-rabbit secondary antibody (1:2000, #ab6721) for 50 min. The density of the protein bands was quantified using Alphalmager™ 2000 Imaging System (Alpha Innotech, San Leandro, USA).

### Statistical analysis

All experiments in this study were performed in triplicate and repeated three times. All data are shown as mean ± standard deviation. The statistical significance was analyzed using two-tailed Student’s t-test or one-way ANOVA (SPSS software, version 24.0, Chicago, USA). A value of *p* < 0.05 (indicated by * or #) was considered statistically significant. * *p* < 0.05, ** *p* < 0.01; # *p* < 0.05, ## *p* < 0.01. Survival data was analyzed using the Kaplan-Meier method. The linear correlation coefficient was used to estimate the correlation in TUG1 mRNA expression vs. miR-34b-5p level and miR-34b-5p level vs. GAB1 mRNA expression.

## Results

### TUG1 was downregulated in mice following CLP operation and induction of TUG1 ameliorated CLP-induced lung injury

**Fig. 1:**
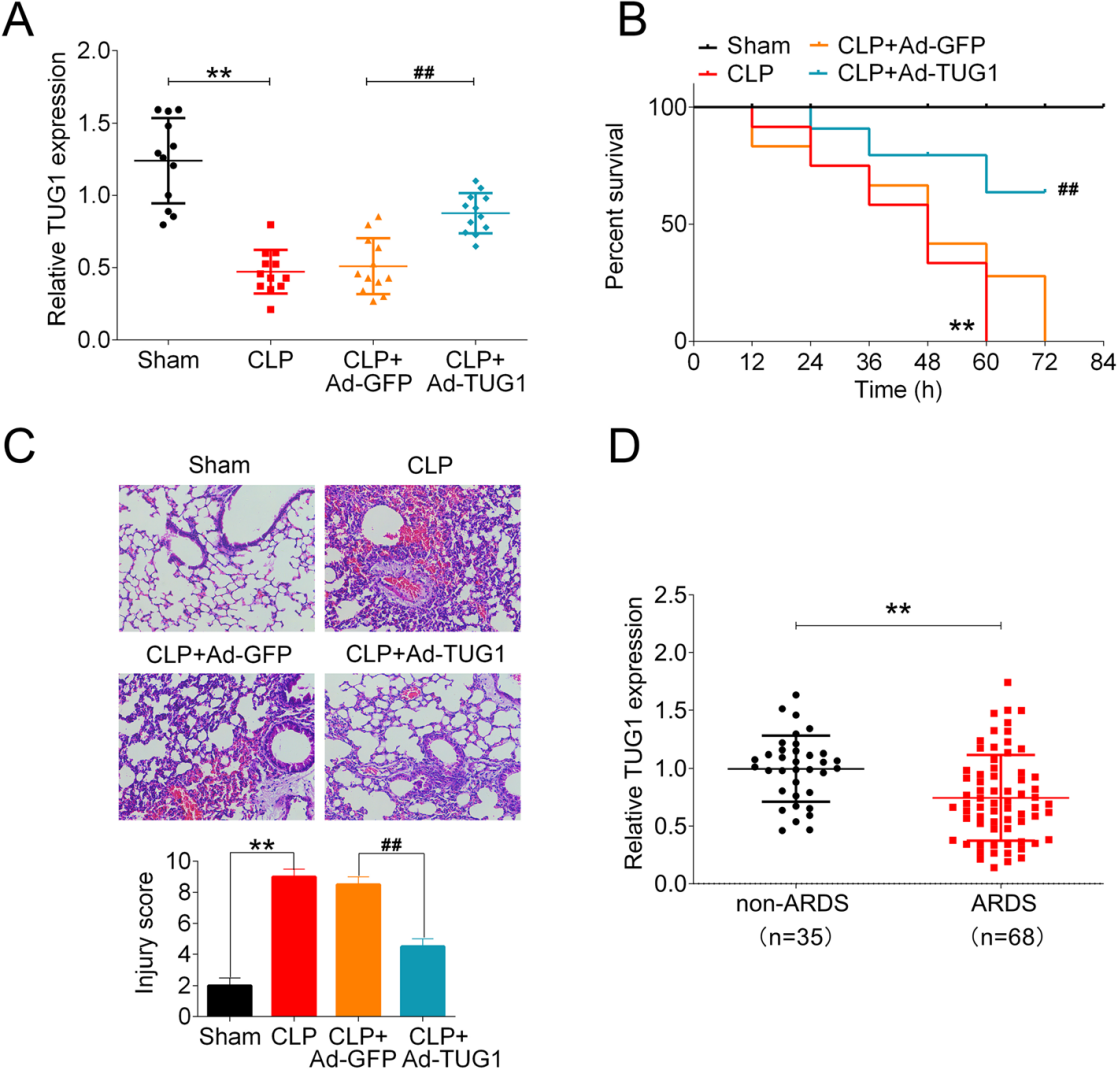
The level of TUG1 in the lung tissues of septic mice and the effect of TUG1 overexpression in sepsis-induced ALI. Adult C57BL/6 mice were randomly assigned into 4 groups: sham, CLP, CLP + Ad-GFP, and CLP + Ad-TUG1 (*n* = 12 per group). CLP, CLP + Ad-GFP, CLP + Ad-TUG1 groups received intravenous injections of vehicle, control adenoviral vector, and adenoviral vector expressing TUG1, respectively, followed by the CLP operation. Animals in the sham group underwent the same CLP procedure without the ligation or puncture of the cecum. (**a**) The expression of TUG1 in mouse lung tissues were examined using qRT-PCR. (**b)** Survival rate of mice within 72 h following CLP procedure. (**c**) Paraffin-embedded lung tissue samples were stained for H&E and the degree of lung damage was determined using lung injury scoring. Representative histological images were shown at 400× magnification. (**d**) The serum level of TUG1 in ARDS patients (*n* = 35) and healthy subjects (*n* = 68) was measured using a ELISA kit

To investigate the regulation role of lncRNA TUG1 in sepsis-induced ALI, CLP surgery was performed on 36 adult male C57BL/6 mice to establish a murine model of sepsis. Compared to sham-operated group, the expression of TUG1 was significantly decreased in mice following CLP surgery. The injection of TUG1-expressing adenoviral vector in CLP-treated mice significantly promoted the level of TUG1 in comparison to that in mice administered of control adenoviral vector (Fig.1a). Mice were monitored for survival after CLP procedure. At the end of the 72 h follow-up period, the survival rate of mice in sham-operated was 100%, whereas CLP operation significantly reduced the survival rate to 0% at 60 h post operation. Mice intravenously injected with control vector (CLP + Ad-GFP group) showed low survival rate with 0% at 72 h post operation, while the induction of TUG1 in CLP + Ad-TUG1 group significantly increased the survival rate following CLP surgery (Fig. 1b). The lung tissues from all groups were collected immediately after death or at the end of the follow-up period, and stained with H&E. As shown in the histological images, sham-operated mice presented normal pulmonary alveoli structure, whereas animals following CLP procedure exhibited collapsed alveolar sacs, thickened alveolar walls, and visible vascular congestion. CLP-induced morphological alterations in the lung tissues were alleviated in mice injected with TUG1-expressing adenoviral vector. The degree of lung tissue damage evaluated by the lung injury scoring also demonstrated that TUG1 overexpression effectively suppressed CLP-induced pulmonary injury (Fig. 1c). In addition, we found that the serum level of TUG1 in ARDS patients was significantly higher than that in healthy subjects (Fig. 1d). These findings suggested that overexpression of TUG1 played a protective role in pulmonary alveoli damage induced by sepsis.

### Overexpression of TUG1 suppressed apoptosis and inflammation in the lung tissues of septic mice

**Fig. 2:**
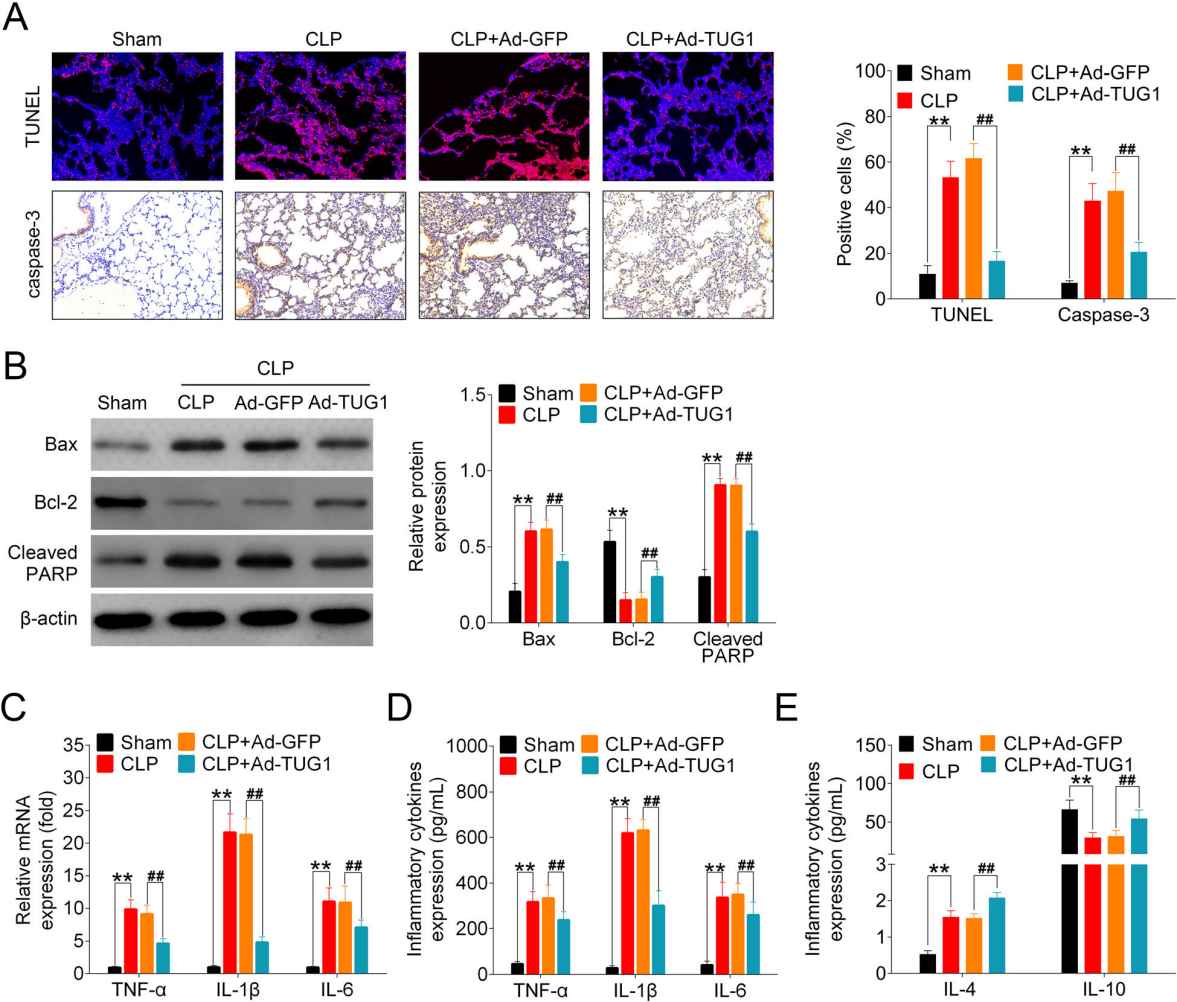
Impact of TUG1 upregulation on CLP-induced apoptosis and inflammation in mouse lung tissues. (**a**) Histological analysis of sectioned mouse lung tissue samples using TUNEL staining and immunohistochemistry for caspase-3 expression. Representative histological images (400× magnification) and the percentage of positively stained cells were shown. (**b**) The levels of Bax, Bcl-2, and cleaved PARP in the lung tissues of all mice were analyzed using Western blot. Representative plots (left) and bar charts (right) were shown. (**c**-**d**) The mRNA and proteins expressions of TNF-α, IL-1β and IL-6 in all animals were detected using qRT-PCR and ELISA, respectively. (**e**) The protein expressions of IL-4 and IL-10 in mouse lung tissues were measured using ELISA

Extensive apoptosis of alveolar epithelial cells has been widely reported in the murine models of ALI [25, 26]. Cytokine-mediated inflammatory process is also a key contributor to the injury of epithelial and endothelial cells in the pathogenesis of ALI [27]. In this study, lung tissue sections were stained with TUNEL reagent for the examination of apoptosis. CLP-operated mice showed an large number of TUNEL-positive cells compared to sham-treated group with few apoptotic cells detected. Extensive apoptosis was also observed in CLP + Ad-GFP group, whereas the overexpression of TUG1 efficiently decreased the number of apoptotic cells in lung tissues of CLP-operated mice. By staining sectioned lung tissues with caspase-3 antibody, we found that the overproduction of caspase-3 in septic animals was suppressed by the injection of adenoviral vector expressing TUG1 (Fig. 2a). Furthermore, we analyzed the pulmonary expressions of pro-apoptotic proteins (Bax and cleaved PARP) and anti-apoptotic proteins (Bcl-2) in all animals. CLP operation significantly increased the levels of Bax and cleaved PARP, but inhibited the expression of Bcl-2 in mouse lung tissues. Administration of TUG1-expressing adenoviral vector reversed the expressions of both pro- and anti-apoptotic proteins with statistical significance (Fig. 2b). To explore the regulatory effect of TUG1 on CLP-induced inflammation, we assessed the levels of TNF-α, IL-1β and IL-6 using qRT-PCR and ELISA. Our data showed that the expressions of all three proinflammatory cytokines were significantly increased in mice with CLP-induced sepsis compared to sham-treated controls. Injection of TUG1-expressing adenoviral vector in septic mice efficiently suppressed the production of proinflammatory cytokines at both mRNA and protein levels (Fig. 2c-d). Next, we measured the expressions of anti-inflammatory cytokines, IL-4 and IL-10, in all groups of mice. The results showed that CLP operation induced the release of IL-4 but reduced the expression of IL-10 in mouse lung tissues. TUG1 overexpression significantly elevated the levels of IL-4 and IL-10 compared to control-vector-injected mice following CLP surgery (Fig. 2e). The above results indicated that TUG1 alleviated sepsis-induced apoptosis and inflammatory responses in CLP-operated mice.

### TUG1 overexpression reduced LPS-induced apoptosis and inflammation in PMVECs

**Fig. 3:**
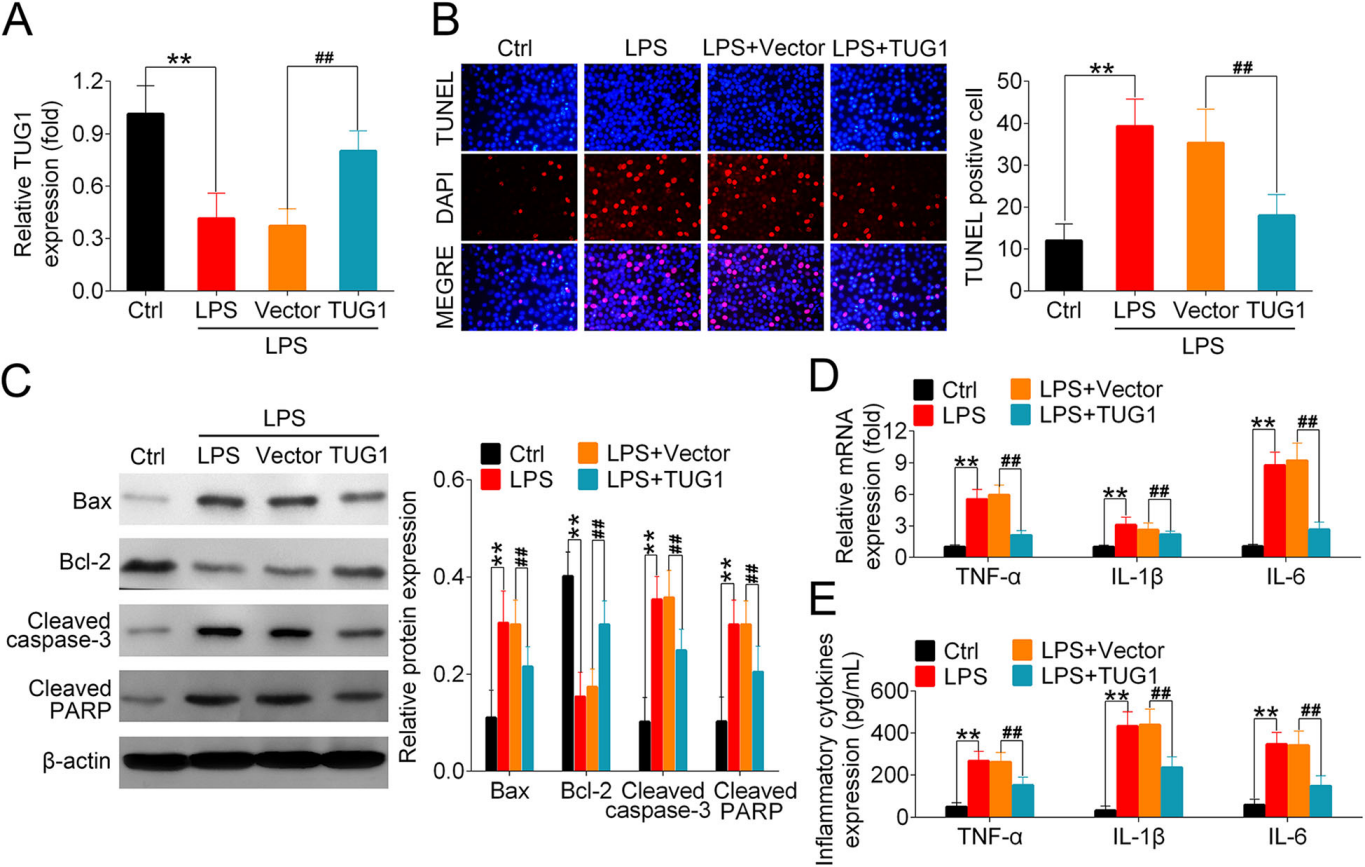
Overexpression of TUG1 mediated the levels of apoptotic markers and proinflammatory cytokines in LPS-treated PMVECs. PMVECs were divided into 4 groups: control (Ctrl), LPS, Vector, and TUG1. LPS, Vector, and TUG1 groups were cultured with serum-free medium containing nothing, control adenoviral vector, and adenoviral vector expressing TUG1, respectively, followed by the 6-h stimulation of 100 ng/mL LPS. Ctrl group remained untreated. (**a**) Relative expression of TUG1 were measured using qRT-PCR 6 h after LPS treatment. (**b**) TUNEL and DAPI staining were performed to detect apoptotic cell death in PMVECs. The number of TUNEL-positive cells were counted in six randomly selected fields per slide. (**c**) The expression of Bax, Bcl-2, cleaved caspase-3, and cleaved PARP in all groups of PMVECs were examined using Western blot. Representative plots (left) and bar charts (right) were shown. (**d**-**e**) The mRNA and proteins levels of TNF-α, IL-1β and IL-6 in PMVECs were assessed using qRT-PCR and ELISA, respectively

To further ascertain the beneficial effect of TUG1 overexpression on sepsis-induced lung damage, we established an in vitro PMVECs cell model of to mimic in vivo septic epithelium via LPS stimulation. LPS-treated cells showed a significant reduction in TUG1 expression in comparison to the control group. The delivery of TUG1 expressing adenoviral vectors prior to LPS treatment significantly increased the level of TUG1 in PMVECs (Fig. 3a). TUNEL staining results demonstrated that numerous apoptotic cells appeared in the wild-type and control vector-transfected PMVECs following LPS stimulation compared to untreated group. However, overexpression of TUG1 significantly decreased the number of TUNEL-positive cells in LPS-challenged PMVECs (Fig. 3b). We then analyzed the levels of apoptotic markers in all PMVECs groups. In consistent with the data shown in mouse septic model, LPS stimulation led to a significant induction of pro-apoptotic proteins, including Bax, cleaved caspase-3 and cleaved PARP, but inhibited the level of anti-apoptotic protein Bcl-2. The transfection of PMVECs with TUG1-expressing vectors successfully restored the expression of Bcl-2 and downregulated the level of these pro-apoptotic proteins (Fig. 3c). The secretion of TNF-α, IL-1β and IL-6 was significantly induced by LPS stimulation in both mRNA and protein levels. By delivering TUG1-expressing vector, the upregulated expressions of proinflammatory cytokines were prominently inhibited in PMVECs compared with control vector-transfected cells (Fig. 3d, e). These data suggested that TUG1 also exerted an anti-apoptotic and anti-inflammatory effect in vitro.

### TUG1 reversely regulated the expression of miR-34b-5p in the models of sepsis

**Fig. 4:**
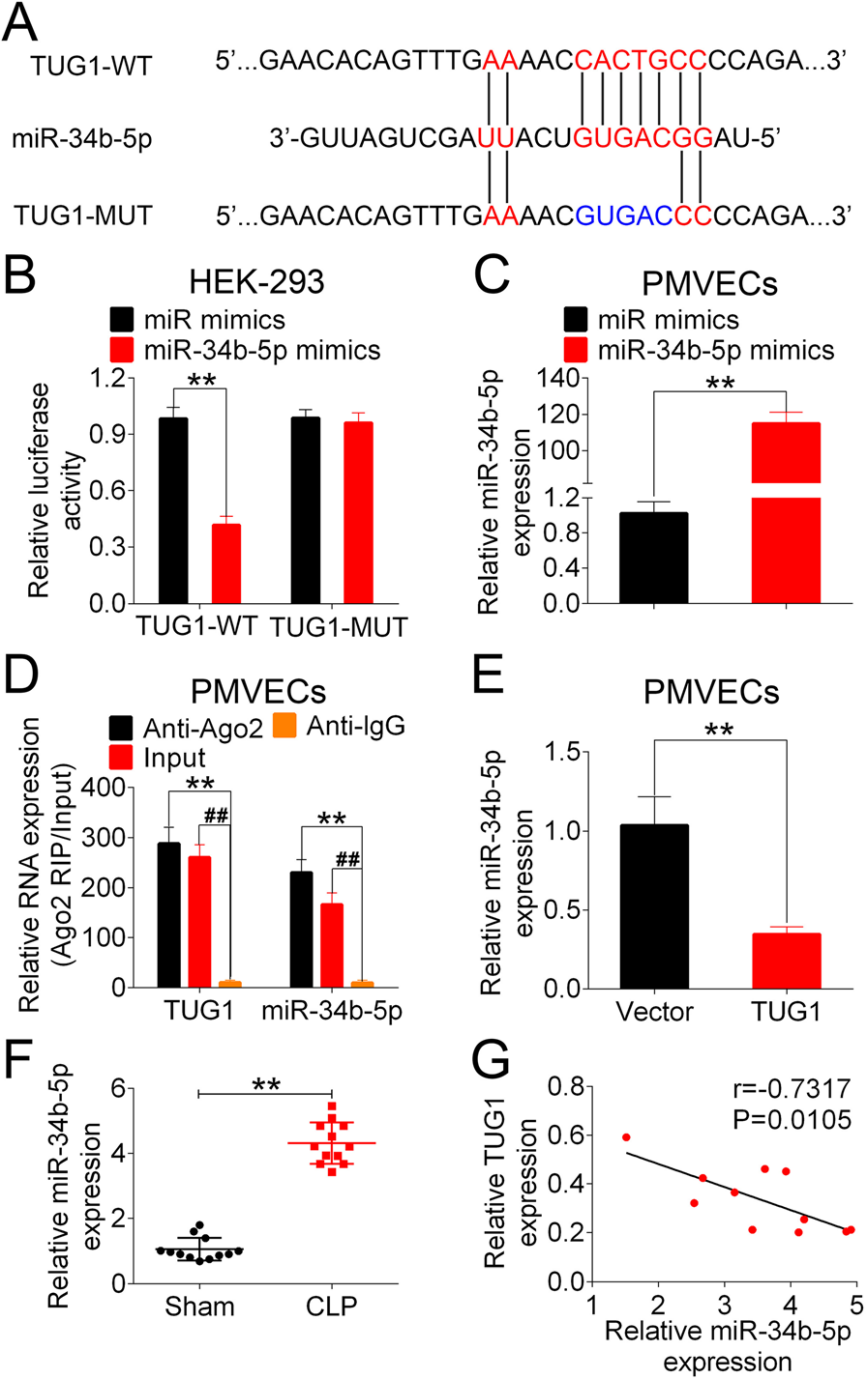
TUG1 targeted on miR-34b-5p in the models of sepsis. (**a**) The putative binding site between TUG1 and miR-34b-5p were predicted on miRDB. The wild-type binding sequence and the designed mutant sequence were indicated as TUG1-WT and TUG1-MUT, respectively. (**b**) PMVECs were transfected with miR mimics or miR-34b-5p mimics. The transfection efficacy was determined by measuring the level of miR-34b-5p in transfected cells. (**c**) HEK-293 T cells were co-transfected with miR mimics (or miR-34b-5p mimics) and TUG1-WT (or TUG1-MUT). The luciferase activity was measuring 48 h post transfection using dual-luciferase reporter assay. (**d**) The RIP assay was performed in PMVECs using a mouse monoclonal anti-Ago2 antibody as a positive control and an anti-IgG antibody as a negative control. The expressions of TUG1 and miR-34b-5p in RISCs were analyzed using qRT-PCR. (**e**) The relative expression of miR-34b-5p in PMVECs transfected with control adenoviral vector (Vector) or adenoviral vector expressing TUG1(TUG1) were examined using qRT-PCR. (**f**) The relative expression of miR-34b-5p in sham- and CLP-operated mice were detected using qRT-PCR. (**g**) The correlation between miR-34b-5p expression and TUG1 level in mouse lung tissues were analyzed using Spearman’s rank correlation test

Next, we searched for the downstream target of TUG1 using the bioinformatic prediction database miRDB. The prediction analysis indicated the miR-34b-5p was a potential target for TUG1 with a putative binding site (Fig. 4a). PMVECs were transfected with miR-34b-5p or the control mimics, followed by the examination of miR-34b-5p expression. PMVECs transfected with miR-34b-5p showed significantly higher level of miR-34b-5p compared to the controls (Fig. 4b). Luciferase reporter assay results showed that HEK-293 T cells co-transfected with miR-34b-5p and TUG1-WT had significantly lower luciferase activity compared to the ones containing miR mimics and TUG1-WT. However, no evident change on the luciferase activity was observed in cells transfected with a mutant TUG1 binding sequence (Fig. 4c). Ago2 protein is an essential component of the RNA-induced silencing complex (RISC) [28]. The RIP assay using Ago2-sepcific antibody allows for the detection of a Ago2-binding RNA and its target for gene silencing [29]. In PMVECs, both TUG1 and miR-34b-5p were enriched in Ago2 immunoprecipitation relative to control IgG, suggesting the interaction between them (Fig. 4d). Compared to the transfection of control vectors, the delivery of TUG1-expressing adenoviral vectors significantly reduced the expression of miR-34b-5p in PMVECs (Fig. 4e). In t mouse lung tissue samples, we found that the level of miR-34b-5p was remarkably higher in CLP-treated mice than that in sham-operated group (Fig. 4f). In addition, the pulmonary expression of TUG1 was negatively correlated with the level of miR-34b-5p with statistical significance (Fig. 4g). Taken together, these results indicated that TUG1 negatively regulated the expression of its downstream target miR-34b-5p.

### GAB1 is a direct downstream target of miR-34b-5p

**Fig. 5:**
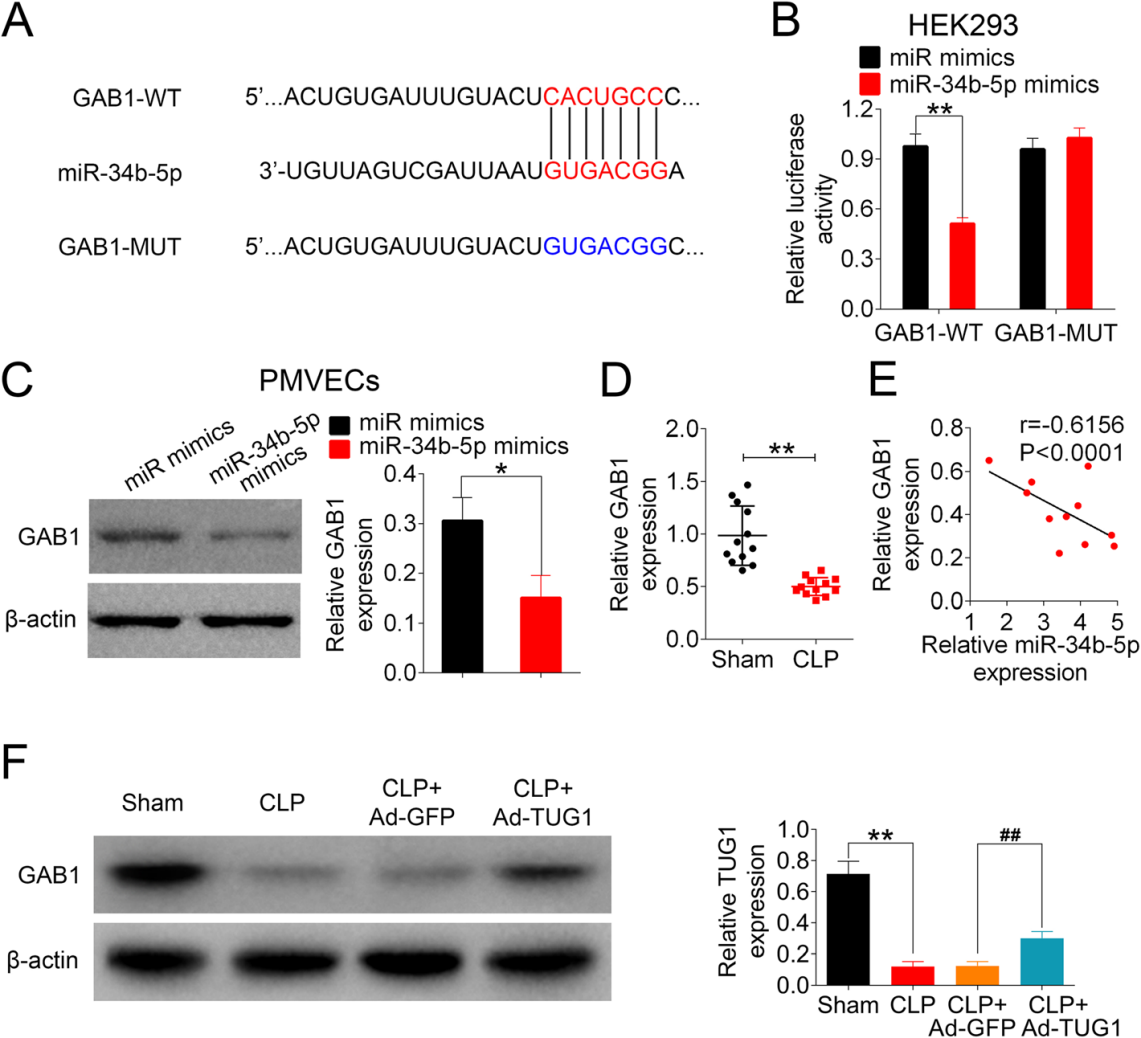
GAB1 is a target gene of miR-34b-5p. (**a**) GAB1 was predicted as a downstream target of miR-34b-5p using TargetScan. The putative GAB1 binding site for miR-34b-5p (GAB1-WT) and the designed mutant sequence (GAB1-MUT) were shown. (**b)** HEK-293 T cells co-transfected with miR mimics (or miR-34b-5p mimics) and GAB1-WT (or GAB1-MUT) were analyzed for luciferase activity 48 h after transfection using dual-luciferase reporter assay. (**c**) PMVECs were transfected with miR mimics or miR-34b-5p mimics. The expression of GAB1 in transfected PMVECs were examined using Western blot. (**d**) The relative expression of GAB1 in sham- and CLP-operated animals were assessed using qRT-PCR. (E) The correlation between miR-34b-5p level and GAB1 expression in mouse lung tissues were examined using Spearman’s rank correlation test. (F) The protein expression of GAB1 in mice was measured using Western blot

To further explore the mechanisms of TUG1/miR-34b-5p-mediated regulation in sepsis-induced ALI, we used TargetScan database to predict a downstream target of miR-34b-5p. GAB1 was shown as a potential target of miR-34b-5p with a putative binding site (Fig. 5a). Dual-luciferase reporter assay showed that the luciferase activity of GAB1-WT was significantly lower in cells transfected with miR-34b-5p mimics in comparison to control mimics cells. On the contrary, cells transfected with GAB1-MUT reporter had no difference on the luciferase activity between miR-34b-5p mimics and control mimics groups (Fig. 5b). By transfecting PMVECs with miR-34b-5p mimics (or miR mimics), we found that the level of GAB1 was significantly decreased in cells overexpressing miR-34b-5p compared to the controls (Fig. 5c). In the animal model, mice following CLP surgery demonstrated significantly lower level of GAB1 in the lung tissues compared the sham group (Fig. 5d). We also found a strong negative correlation between miR-34b-5p level and GAB1 expression in vivo with statistical significance (Fig. 5e). The injection of TUG1 adenoviral vector partially but significantly restored the expression of GAB1 in CLP-treated mice (Fig. 5f). The above findings suggested that GAB1 was a downstream target of miR-34b-5p.

### TUG1 inhibited LPS-induced apoptosis and inflammation via targeting miR-34b-5p and GAB1

**Fig. 6:**
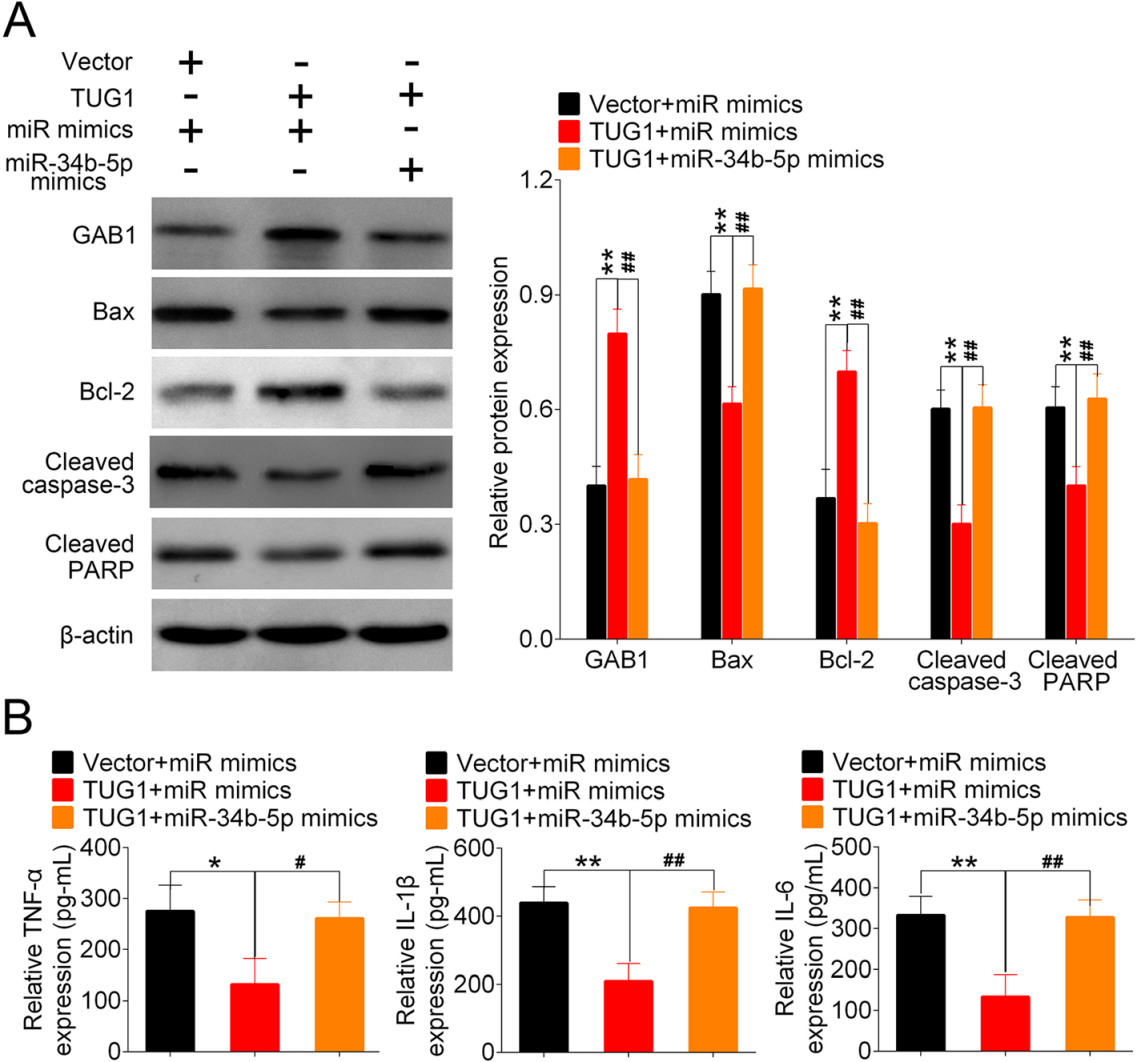
TUG1 regulated sepsis-induced apoptosis and inflammation via the modulation of miR-34b-5p and GAB1. PMVECs were divided into 3 groups: Vector+miR mimics, TUG1 + miR mimics and TUG1 + miR-34b-5p mimics. Cells in Vector+miR mimics group were co-transfected with control adenoviral vector and control mimics. PMVECs in TUG1 + miR mimics and TUG1 + miR-34b-5p mimics groups were co-transfected with adenoviral vector expressing TUG1 and control mimics (or miR-34b-5p mimics). LPS stimulation was performed 24 h post transfection. (**a**) The protein levels of GAB1, Bax, Bcl-2, and cleaved caspas-3, and cleaved PARP in all groups were determined using Western blot. Representative plots (left) and bar charts (right) were shown. (**b**) The production of TNF-α, IL-1β and IL-6 in co-transfected PMVECs were measured using ELISA

Finally, to ascertain TUG1 alleviated sepsis-induced ALI via targeting miR-34b-5pand GAB1, we co-transfected PMVECs with TUG1-expressing vector and miR-34b-5p mimics (or control mimics) before LPS stimulation. Cells transfected with control vector and miR mimics were served as a control group. Protein analysis showed that the overexpression of TUG1 significantly enhanced the expression of GAB1 in PMVECs compared to control cells, whereas the delivery of miR-34b-5p mimics attenuated the upregulation of GAB1 induced by TUG1. TUG1-inibited apoptosis was also effectively restored by the overexpression of miR-34b-5p (Fig. 6a). Moreover, the expressions of TNF-α, IL-1β and IL-6 in PMVECs were significantly reduced by TUG1 overexpression, whereas the level of these proinflammatory cytokines were restored to a normal level by the delivery of miR-34b-5p mimics (Fig. 6b). These data indicated that TUG1 impeded LPS-stimulated apoptosis and inflammation via the suppression of miR-34b-5p and the upregulation of GAB1 in PMVECs.

## Discussion

Sepsis is a serious complication of infection characterized by dysregulated immune responses, microvascular thrombosis and subsequent multiple organ dysfunction, and has become a major etiology of ALI [30]. During the progression of sepsis, the accumulation of endotoxin, inflammatory cytokines and thrombotic factors in the alveoli contributes to the pulmonary edema formation, neutrophil migration, and tissue damage in the lungs [31]. It has been reported that patients with sepsis-induced ALI had higher organ dysfunction, illness severity, and in-hospital mortality rates compared to the ones with non-sepsis-induced ALI [32]. Although the ventilatory management of ALI has a proven survival advantage, the investigations of potential therapeutic treatment prior to the progression of respiratory failure are currently underway [33]. In the present study, we reported that lncRNA TUG1 ameliorated sepsis-induced ALI by the suppression of inflammatory responses and apoptotic activity, via targeting miR-34b-5p and GAB1. These data highlighted the potential therapeutic role of TUG1 in management of sepsis-induced ALI.

TUG1 is a spliced, polyadenylated lncRNA located at chromosome 22q12 [12]. The regulatory roles of TUG1 in the pathogenesis of inflammation-related disorders and the potential underlying mechanisms have been widely discussed. The induction of TUG1 protected interstitial cells of Cajal from TNF-α-induced apoptosis and proinflammatory cytokines expression via downregulating miR-127 [16]. A study in H9c2 cardiomyoblasts found that TUG1 induction alleviated LPS-induced cell injury, manifested by declined apoptosis rate, decreased secretion of pro-apoptotic factors and inflammatory cytokines, by inhibiting the expression of miR-29b [34]. In this study, we found that the level of TUG1 was significantly reduced in mice following CLP surgery compared to the sham-operated group. The injection of TUG1-expressing adenoviral vector in CLP mice, however, alleviated CLP-induced lung tissue damage, accompanied with significantly downregulated inflammatory responses and apoptosis. The anti-apoptotic and anti-inflammatory effects of TUG1 were also observed in PMVECs, in which the LPS-induced secretion of proinflammatory cytokines and apoptosis were efficiently inhibited in cells overexpressing TUG1.

It has been recently uncovered that lncRNA can function as a miRNA sponge to inactivate its target miRNAs [35]. In our study, miR-34b-5p was predicted as a direct downstream target of TUG1 and their expressions were negatively correlated. MiR-34b-5p is a member of miR-34b family which is dominantly expressed in the lungs [36]. Recent evidence suggests that miR-34b-5p is prominently upregulated in inflammation-associated diseases, such as intracranial aneurysm and acute graft-versus-Host disease [37, 38]. Xie et al. showed that inhibition of miR-34b-5p attenuated lung inflammation and the expressions of Bax and cleaved-caspase-3 in mice subjected to LPS stimulation [39]. Here, we showed that TUG1-supressed apoptosis and the expressions of TNF-α, IL-1β and IL-6 in LPS-treated PMVECs were restored by the delivery of miR-34b-5p mimics, implicating the adverse regulatory role of miR-34b-5p in ALI.

We further explored the downstream cascade of TUG1/miR-34b-5p axis involved in the regulation of ALI, and found that GAB1 was directly targeted by miR-34b-5p. GAB1 is a scaffolding protein that belongs to the GRB2-associated binding family [40]. The alveolar epithelium-specific knockout of GAB1 in mice reduced the level of surfactant protein in alveolar type-II cells, promoted LPS-induced pulmonary inflammation, and aggravated bleomycin-triggered fibrotic lung injury, suggesting a vital role of Gab1 in the regulation of alveolar homeostasis [41]. In this study, we found that GAB1 was significantly downregulated in mice following CLP surgery, whereas the injection of TUG1 adenoviral vector significantly recovered the expression of GAB1 in CLP-treated mice. Moreover, in vitro data showed that the production of GAB1 was inversely regulated by miR-34b-5p, but was positively correlated with the expression of TUG1, suggesting the involvement of GAB1 in sepsis-induced ALI.

## Conclusions

Taken together, the expression of lncRNA TUG1 was downregulated in CLP-operated mice. The induction of TUG1 ameliorated sepsis-induced lung injury, the secretion proinflammatory cytokines, and apoptosis via inhibiting miR-34b-5p and promoting GAB1. Our study highlighted the therapeutic potential of TUG1 for the management of sepsis-induced ALI.

## Data Availability

All data generated or analyzed during this study are included in this published article.

## Abbreviations

ALI: Acute lung injury
ARDS: Acute respiratory distress syndrome
Bax: Bcl-2-associated X protein
Bcl-2: B-cell lymphoma 2
CLP: Cecal ligation and puncture
ELISA: Enzyme-linked immunosorbent assay
GAB1: GRB2 associated binding protein 1
H&E: Hematoxylin and eosin
IL: Interleukin
LncRNAs: Long non-coding RNAs
LPS: Lipopolysaccharide
miRNAs: MicroRNAs
PARP: Poly ADP ribose polymerase
PMVECs: Pulmonary microvascular endothelial cells
qRT-PCR: Quantitative real-time PCR
RIP: RNA-binding protein immunoprecipitation
RISC: RNA-induced silencing complex
TNF-α: Tumor necrosis factor alpha
TUG1: Taurine up-regulated gene 1

## References

[1] Levitt JE, Matthay MA (2010). The utility of clinical predictors of acute lung injury: towards prevention and earlier recognition. Expert Rev Respir Med.

[2] Rubenfeld GD, Caldwell E, Peabody E, Weaver J, Martin DP, Neff M, Stern EJ, Hudson LD (2005). Incidence and outcomes of acute lung injury. N Engl J Med.

[3] Johnson ER, Matthay MA (2010). Acute lung injury: epidemiology, pathogenesis, and treatment. J Aerosol Med Pulm Drug Deliv.

[4] Meduri GU, Kohler G, Headley S, Tolley E, Stentz F, Postlethwaite A (1995). Inflammatory cytokines in the BAL of patients with ARDS. Persistent elevation over time predicts poor outcome. Chest.

[5] Parsons PE, Eisner MD, Thompson BT, Matthay MA, Ancukiewicz M, Bernard GR, Wheeler AP (2005). Lower tidal volume ventilation and plasma cytokine markers of inflammation in patients with acute lung injury. Crit Care Med.

[6] Martin TR, Nakamura M, Matute-Bello G (2003). The role of apoptosis in acute lung injury. Crit Care Med.

[7] Chen LL, Carmichael GG (2010). Long noncoding RNAs in mammalian cells: what, where, and why?. Wiley Interdiscip Rev RNA.

[8] Wilusz JE, Sunwoo H, Spector DL (2009). Long noncoding RNAs: functional surprises from the RNA world. Genes Dev.

[9] Mathy NW, Chen XM (2017). Long non-coding RNAs (lncRNAs) and their transcriptional control of inflammatory responses. J Biol Chem.

[10] Reddy MA, Chen Z, Park JT, Wang M, Lanting L, Zhang Q, Bhatt K, Leung A, Wu X, Putta S (2014). Regulation of inflammatory phenotype in macrophages by a diabetes-induced long noncoding RNA. Diabetes.

[11] Zhu X, Du J, Yu J, Guo R, Feng Y, Qiao L, Xu Z, Yang F, Zhong G, Liu F (2019). LncRNA NKILA regulates endothelium inflammation by controlling a NF-kappaB/KLF4 positive feedback loop. J Mol Cell Cardiol.

[12] Young TL, Matsuda T, Cepko CL (2005). The noncoding RNA taurine upregulated gene 1 is required for differentiation of the murine retina. Curr Biol : CB.

[13] Zhang Q, Geng PL, Yin P, Wang XL, Jia JP, Yao J (2013). Down-regulation of long non-coding RNA TUG1 inhibits osteosarcoma cell proliferation and promotes apoptosis. Asian Pac J Cancer Prev : APJCP.

[14] Han Y, Liu Y, Gui Y, Cai Z (2013). Long intergenic non-coding RNA TUG1 is overexpressed in urothelial carcinoma of the bladder. J Surg Oncol.

[15] Niu Y, Ma F, Huang W, Fang S, Li M, Wei T, Guo L (2017). Long non-coding RNA TUG1 is involved in cell growth and chemoresistance of small cell lung cancer by regulating LIMK2b via EZH2. Mol Cancer.

[16] Su S, Liu J, He K, Zhang M, Feng C, Peng F, Li B, Xia X (2016). Overexpression of the long noncoding RNA TUG1 protects against cold-induced injury of mouse livers by inhibiting apoptosis and inflammation. FEBS J.

[17] National Research Council Institute for Laboratory Animal R. In: Guide for the Care and Use of Laboratory Animals. edn. Washington (DC): National Academies Press (US).Copyright 1996 by the National Academy of Sciences. All rights reserved.; 1996.

[18] Matsuda N, Hattori Y, Jesmin S, Gando S (2005). Nuclear factor-kappaB decoy oligodeoxynucleotides prevent acute lung injury in mice with cecal ligation and puncture-induced sepsis. Mol Pharmacol.

[19] Ruiz S, Vardon-Bounes F, Merlet-Dupuy V, Conil JM, Buleon M, Fourcade O, Tack I, Minville V (2016). Sepsis modeling in mice: ligation length is a major severity factor in cecal ligation and puncture. Intensive Care Med Exp.

[20] Lee JH, Song MY, Song EK, Kim EK, Moon WS, Han MK, Park JW, Kwon KB, Park BH (2009). Overexpression of SIRT1 protects pancreatic beta-cells against cytokine toxicity by suppressing the nuclear factor-kappaB signaling pathway. Diabetes.

[21] Matute-Bello G, Downey G, Moore BB, Groshong SD, Matthay MA, Slutsky AS, Kuebler WM (2011). An official American Thoracic Society workshop report: features and measurements of experimental acute lung injury in animals. Am J Respir Cell Mol Biol.

[22] World Medical Association Declaration of Helsinki: ethical principles for medical research involving human subjects. Jama 2013, 310(20):2191–2194. 10.1001/jama.2013.281053 24141714

[23] Hebbel RP, Vercellotti GM, Pace BS, Solovey AN, Kollander R, Abanonu CF, Nguyen J, Vineyard JV, Belcher JD, Abdulla F (2010). The HDAC inhibitors trichostatin a and suberoylanilide hydroxamic acid exhibit multiple modalities of benefit for the vascular pathobiology of sickle transgenic mice. Blood.

[24] Xu J, Xu Y (2017). The lncRNA MEG3 downregulation leads to osteoarthritis progression via miR-16/SMAD7 axis. Cell & bioscience.

[25] Kitamura Y, Hashimoto S, Mizuta N, Kobayashi A, Kooguchi K, Fujiwara I, Nakajima H (2001). Fas/FasL-dependent apoptosis of alveolar cells after lipopolysaccharide-induced lung injury in mice. Am J Respir Crit Care Med.

[26] Kawasaki M, Kuwano K, Hagimoto N, Matsuba T, Kunitake R, Tanaka T, Maeyama T, Hara N (2000). Protection from lethal apoptosis in lipopolysaccharide-induced acute lung injury in mice by a caspase inhibitor. Am J Pathol.

[27] Goodman RB, Pugin J, Lee JS, Matthay MA (2003). Cytokine-mediated inflammation in acute lung injury. Cytokine Growth Factor Rev.

[28] Beitzinger M, Peters L, Zhu JY, Kremmer E, Meister G (2007). Identification of human microRNA targets from isolated argonaute protein complexes. RNA Biol.

[29] Chi SW, Zang JB, Mele A, Darnell RB (2009). Argonaute HITS-CLIP decodes microRNA-mRNA interaction maps. Nature.

[30] Sagy M, Al-Qaqaa Y, Kim P (2013). Definitions and pathophysiology of sepsis. Current problems in pediatric and adolescent health care.

[31] Toner P, McAuley DF, Shyamsundar M (2015). Aspirin as a potential treatment in sepsis or acute respiratory distress syndrome. Crit Care (London, England).

[32] Sevransky JE, Martin GS, Shanholtz C, Mendez-Tellez PA, Pronovost P, Brower R, Needham DM (2009). Mortality in sepsis versus non-sepsis induced acute lung injury. Crit Care (London, England).

[33] Levitt JE, Matthay MA (2012). Clinical review: Early treatment of acute lung injury--paradigm shift toward prevention and treatment prior to respiratory failure. Crit Care (London, England).

[34] Zhang H, Li H, Ge A, Guo E, Liu S, Zhang L (2018). Long non-coding RNA TUG1 inhibits apoptosis and inflammatory response in LPS-treated H9c2 cells by down-regulation of miR-29b. Biomed Pharmacother = Biomed Pharmacother.

[35] Paraskevopoulou MD, Hatzigeorgiou AG (2016). Analyzing MiRNA-LncRNA Interactions. Methods Mol Biol (Clifton, NJ).

[36] Liang Y, Ridzon D, Wong L, Chen C (2007). Characterization of microRNA expression profiles in normal human tissues. BMC Genomics.

[37] Li L, Sima X, Bai P, Zhang L, Sun H, Liang W, Liu J, Zhang L, Gao L (2012). Interactions of miR-34b/c and TP53 polymorphisms on the risk of intracranial aneurysm. Clin Dev Immunol.

[38] Jalapothu D, Boieri M, Crossland RE, Shah P, Butt IA, Norden J, Dressel R, Dickinson AM, Inngjerdingen M (2016). Tissue-specific expression patterns of MicroRNA during acute graft-versus-host disease in the rat. Front Immunol.

[39] Xie W, Lu Q, Wang K, Lu J, Gu X, Zhu D, Liu F, Guo Z (2018). miR-34b-5p inhibition attenuates lung inflammation and apoptosis in an LPS-induced acute lung injury mouse model by targeting progranulin. J Cell Physiol.

[40] Holgado-Madruga M, Emlet DR, Moscatello DK, Godwin AK, Wong AJ (1996). A Grb2-associated docking protein in EGF- and insulin-receptor signalling. Nature.

[41] Wang K, Qin S, Liang Z, Zhang Y, Xu Y, Chen A, Guo X, Cheng H, Zhang X, Ke Y (2016). Epithelial disruption of Gab1 perturbs surfactant homeostasis and predisposes mice to lung injuries. Am J Physiol Lung Cell Mol Physiol.

